# Change in salivary cortisol levels in children (aged 9–12 years) with obesity and respiratory diseases during a 28-day spa treatment: a pilot prospective study

**DOI:** 10.1007/s00431-025-06723-3

**Published:** 2026-01-16

**Authors:** Z. Třískala, D. Jandová, M. Hill, M. Bičíková, L. Máčová

**Affiliations:** 1 Charles University in Prague, Faculty of Medicine in Plzen, Jahodová 2889/42, Záběhlice, 106 00. Praha 10, Hlavní město, Prague, Czech Republic; 2 Institute of Endocrinology, Department of Steroids and Proteofactors, Prague, Czech Republic; 3 Institute for Postgraduate Medical Education (IPVZ), Prague, Czech Republic

**Keywords:** Salivary cortisol, Children, Obesity, Respiratory diseases, Balneotherapy, HPA axis, Biomarkers

## Abstract

Evidence on the neuroendocrine effects of pediatric spa treatment remains limited. Salivary cortisol is a validated, non-invasive biomarker of hypothalamic–pituitary–adrenal (HPA) axis activity, which may serve as an indicator of physiological adaptation to structured environmental and behavioral interventions. To determine whether a standardized 28-day spa treatment is associated with changes in morning salivary cortisol in children aged 9–12 years with obesity or chronic respiratory diseases, and to compare cortisol trajectories in these diagnostic groups with those of healthy controls. This prospective observational study included 95 children undergoing spa treatment and 38 school-based healthy controls assessed 24 days apart. Morning saliva (07:45–08:30) was analyzed using a validated LC–MS/MS method. Cortisol was successfully measured in 34 of 36 children with obesity, 18 of 50 children with respiratory diseases not receiving corticosteroids, and 36 of 38 healthy controls. Somatic and functional assessments were performed across the clinical cohort. Children with obesity showed a significant decrease in morning salivary cortisol (3.32 → 2.36 nmol/L; mean change − 0.96 nmol/L; *p* = 0.004; *d* =  − 0.54). Children with respiratory diseases showed a mild, non-significant increase (2.68 → 3.48 nmol/L; + 0.80 nmol/L; *p* = 0.057; *d* = 0.47). Healthy controls showed a small increase (3.52 → 4.02 nmol/L; + 0.50 nmol/L; *p* = 0.089; *d* = 0.25). Cortisol trajectories were broadly consistent with functional improvements within the spa cohort. Between-group differences should be interpreted in the context of natural variability and environmental factors influencing the school-based control population.

*Conclusions:* Diagnosis-dependent changes in morning salivary cortisol were observed during a 28-day spa treatment program. The decrease in children with obesity and the mild increase in those with respiratory diseases may indicate differing patterns of physiological adaptation. These findings support the feasibility of incorporating salivary cortisol into future controlled pediatric trials aimed at evaluating neuroendocrine responses to spa therapy.

**Table:** 

**What is Known:**
• *Salivary cortisol is a validated, non-invasive biomarker of hypothalamic–pituitary–adrenal (HPA) axis activity and is widely used in pediatric research on stress regulation*. • *Spa treatment (balneotherapy) combines environmental and behavioral components, yet biomarker-based studies have been conducted almost exclusively in adults. Evidence from pediatric spa populations is currently lacking*.
**What is New:**
• *This pilot study provides the first pediatric data on morning salivary cortisol measured before and after a structured 28-day spa treatment program*. • *These findings offer initial effect-size estimates and suggest that morning salivary cortisol may be feasible to incorporate as a non-invasive marker in future controlled pediatric studies assessing physiological adaptation during spa treatment*.

• *Salivary cortisol is a validated, non-invasive biomarker of hypothalamic–pituitary–adrenal (HPA) axis activity and is widely used in pediatric research on stress regulation*.

• *Spa treatment (balneotherapy) combines environmental and behavioral components, yet biomarker-based studies have been conducted almost exclusively in adults. Evidence from pediatric spa populations is currently lacking*.

• *This pilot study provides the first pediatric data on morning salivary cortisol measured before and after a structured 28-day spa treatment program*.

• *These findings offer initial effect-size estimates and suggest that morning salivary cortisol may be feasible to incorporate as a non-invasive marker in future controlled pediatric studies assessing physiological adaptation during spa treatment*.

## Introduction

Salivary cortisol is a widely used non-invasive biomarker of hypothalamic–pituitary–adrenal (HPA) axis activity and stress regulation in children [1–3]. Morning cortisol levels can reflect both chronic stress exposure and short-term physiological adaptation and are sensitive to lifestyle, environmental changes, physical activity, and chronic disease states [1, 2]. Children with obesity or chronic respiratory diseases have been shown to exhibit altered HPA-axis reactivity, which may have implications for metabolic health, immune function, and psychological well-being [4–6].

Despite the recognized potential of salivary cortisol in pediatric research, very little is known about its behavior in the context of structured therapeutic or environmental interventions. Spa treatment, common in Central Europe, combines physical activity, regimented routines, dietary modification, and exposure to natural climatic stimuli. These multicomponent interventions may influence stress physiology, yet pediatric evidence remains scarce. Most existing studies evaluating hormonal or metabolic changes during spa therapy have been conducted exclusively in adults. For example, a study from the Priessnitz Spa in Jeseník demonstrated measurable alterations in corticosteroid metabolome profiles in adults undergoing a structured rehabilitation program, suggesting the potential for balneotherapy to modulate steroid hormone pathways [7]. However, no comparable biomarker studies have been performed in pediatric spa populations.

This lack of pediatric data represents a clear gap in current knowledge. From a clinical perspective, there is a growing need for objective, minimally invasive markers capable of capturing treatment-related adaptation in children undergoing complex rehabilitation programs, where subjective outcomes and heterogeneous diagnoses often complicate evaluation. Given the non-invasive nature of salivary cortisol sampling and the relevance of monitoring HPA-axis regulation in children [1–3], it is important to determine whether spa therapy in childhood is associated with measurable neuroendocrine adaptation.

This pilot study aims to assess whether a standardized 28-day spa treatment is associated with changes in morning salivary cortisol in children aged 9–12 years with obesity and chronic respiratory diseases, compared to school-based healthy controls. A secondary aim is to examine whether hormonal changes correspond with alterations in somatic and functional parameters such as BMI, spirometry, and six-minute walk distance [8–10]. By providing preliminary effect-size estimates (Cohen’s *d*) [11–13] and feasibility data, this study seeks to inform the design of future controlled trials evaluating neuroendocrine adaptation to spa treatment in pediatric populations.

## Methods

### Study design

This study was a longitudinal, non-randomized, prospective observational project evaluating the effects of a standardized 28-day spa treatment on neuroendocrine, anthropometric, and functional parameters in children aged 9–12 years. Assessments were performed within the first days after admission to the spa facilities and repeated after 21–26 days. Healthy controls underwent the same assessments with a 24-day interval. The study was conducted in 2022, following earlier pilot work performed in 2020–2021.

### Participants

A total of 440 children were screened. Ninety-five children met the inclusion criteria and were enrolled into the spa-treatment cohort. Inclusion criteria were as follows: age 9–12 years; absence of neurological abnormalities (e.g., balance disorders, abnormal Romberg test, nystagmus); no chronic hormonal therapy (e.g., systemic or inhaled corticosteroids, thyroid hormone replacement); absence of menarche in girls; BMI > 12 kg/m^2^; and no acute complications during treatment. The control group consisted of 38 healthy school-based children.

### Diagnostic classification

Among spa-treated children, 50 had chronic respiratory diseases (predominantly bronchial asthma), 36 had obesity, 5 had urinary tract disorders, and 4 had atopic eczema. Children receiving systemic or inhaled corticosteroids (*n* = 33) were excluded from cortisol analyses.

Morning salivary cortisol at both time points was successfully measured in 34 of 36 children with obesity, 18 of 50 with respiratory diseases, 6 of 9 children with other diagnoses, and 36 of 38 healthy controls.

### Salivary cortisol collection and analysis

Morning saliva (07:45–08:30) was collected using Salivette swabs. In the spa cohort, samples were obtained before breakfast and tooth brushing under fasting and hydration instructions (water only). In the control group, samples were collected within the same time window, although full standardization of morning routines was not feasible due to the school environment.

Samples were centrifuged and frozen at − 20 °C and transported to the Institute of Endocrinology in Prague. After thawing and vortex-mixing, 500 µL of saliva was extracted with 2 mL diethyl ether (G.R., Lach-Ner, Neratovice, Czech Republic). The aqueous phase was frozen on dry ice, and the organic phase decanted. The extract was evaporated under nitrogen and reconstituted in 50 µL of physiological saline.

Cortisol was quantified using the IVD-certified ClinMass® LC–MS/MS Steroid Panel (RECIPE Chemicals + Instruments GmbH, Munich, Germany), validated for saliva extracts. Chromatographic separation was performed on an ExionLC AD system, and detection on a QTRAP® 6500 + tandem mass spectrometer (Sciex, Framingham, MA, USA). Data were processed using Analyst® software (Sciex).

Analytical performance characteristics were as follows: lower limit of quantification 0.5 nmol/L; linearity 0.5–100 nmol/L; intra-assay CV 4.3%; inter-assay CV 6.7% (year of analysis: 2022).

### Somatic and functional assessments

Anthropometric measurements included height, weight, BMI, chest expansion, waist and hip circumference, limb circumference, and body composition assessed via bioimpedance (TANITA BC-100, GMON software).

Spirometry was performed using a portable ultrasonic spirometer (SpiroSonic FLO) according to ATS/ERS standards [8]. Physical fitness was assessed with the Six-Minute Walk Test (6MWT) [9]. Motor coordination and agility were assessed using the Four Square Step Test (FSST), a validated tool for dynamic balance assessment [10]. Musculoskeletal status was evaluated using the Computer Kinesiology® system (CKRL score).

### Statistical analysis

Continuous variables were transformed using power transformations to improve symmetry and homoscedasticity [11–13]. ANOVA models included the factors Subj (GDM), GDM × Stage, and Dgmain, as specified in the statistical protocol. Linear regression diagnostics followed Meloun et al. [11–13]. Least significant difference (LSD) was used for post hoc comparisons.

Numerical results are reported as sample size (*n*), mean ± SD at baseline and follow-up, paired mean change with 95% CI, exact *p*-value, and effect size (Cohen’s *d*). Sex-stratified analyses were performed using paired *t*-tests. Statistical significance was defined as *α* = 0.05.

### Ethical approval

The study was approved by the Ethics Committee of the Institute of Endocrinology, Prague. Written informed consent was obtained from legal guardians and from participating children. The study adhered to the Declaration of Helsinki.

## Results

Unless otherwise specified, results are reported as *n*, mean ± SD at baseline and follow-up, paired mean change with 95% CI, exact *p*-value, and within-subject effect size (Cohen’s *d*). Detailed baseline characteristics across diagnostic groups are presented in Table 1. Post-treatment anthropometric and functional characteristics are shown in Table 2.

**Table 1:** Baseline characteristics of children across diagnostic groups (Stage 1)

Group	Age (years)	BMI (kg/m^2^)	6MWT distance (m)	FSST time (s)	Salivary cortisol (nmol/L)	CKRL score
OB	10.86 ± 1.13 (*n* = 36)	29.42 ± 4.44 (*n* = 36)	515.03 ± 71.04 (*n* = 36)	8.99 ± 2.52 (*n* = 36)	3.31 ± 2.05 (*n* = 35)	90.81 ± 10.66 (*n* = 36)
RD	10.41 ± 1.32 (*n* = 50)	19.78 ± 3.78 (*n* = 50)	628.18 ± 72.56 (*n* = 50)	9.32 ± 3.47 (*n* = 50)	2.90 ± 0.97 (*n* = 20)	82.61 ± 10.73 (*n* = 50)
OTHER	10.22 ± 1.09 (*n* = 9)	18.76 ± 2.54 (*n* = 9)	616.11 ± 58.94 (*n* = 9)	9.10 ± 2.78 (*n* = 9)	4.98 ± 3.07 (*n* = 7)	82.44 ± 12.96 (*n* = 9)
HC	10.15 ± 1.04 (*n* = 38)	18.89 ± 3.22 (*n* = 38)	—	8.70 ± 2.10 (*n* = 38)	3.58 ± 2.99 (*n* = 37)	80.56 ± 9.77 (*n* = 38)
Total	10.44 ± 1.20 (*n* = 133)	22.06 ± 5.85 (*n* = 133)	583.69 ± 88.80 (*n* = 95)	9.03 ± 2.81 (*n* = 133)	3.44 ± 2.40 (*n* = 99)	84.22 ± 11.25 (*n* = 133)

*HC* = healthy controls; *OB* = obesity; OTHER = dermatology + nephrology/BD; *RD* = respiratory diseases; *CKRL* = cumulative dysfunction score from the Computer Kinesiology system. Some *n* values differ between variables due to missing measurements (e.g., incomplete cortisol sampling or unavailable CKRL assessment)

**Table 2:** Post-treatment characteristics of children across diagnostic groups (Stage 2)

Group	Age (years)	BMI (kg/m^2^)	6MWT distance (m)	FSST time (s)	Salivary cortisol (nmol/L)	CKRL score
OB	10.94 ± 1.15 (*n* = 36)	27.62 ± 4.33 (*n* = 36)	545.03 ± 77.73 (*n* = 36)	7.50 ± 1.88 (*n* = 36)	2.35 ± 1.26 (*n* = 35)	74.75 ± 6.79 (*n* = 36)
RD	10.50 ± 1.32 (*n* = 50)	19.29 ± 3.78 (*n* = 50)	648.63 ± 58.33 (*n* = 50)	7.99 ± 3.12 (*n* = 50)	3.89 ± 1.99 (*n* = 20)	77.83 ± 9.93 (*n* = 50)
OTHER	10.20 ± 1.14 (*n* = 9)	18.53 ± 2.47 (*n* = 9)	661.11 ± 79.53 (*n* = 9)	7.44 ± 1.01 (*n* = 9)	6.01 ± 5.16 (*n* = 7)	74.40 ± 7.35 (*n* = 9)
HC	10.31 ± 1.03 (*n* = 38)	19.14 ± 3.31 (*n* = 38)	—	8.13 ± 1.93 (*n* = 38)	4.14 ± 2.46 (*n* = 37)	78.59 ± 7.36 (*n* = 38)
Total	10.54 ± 1.20 (*n* = 133)	21.44 ± 5.29 (*n* = 133)	610.36 ± 85.45 (*n* = 95)	9.03 ± 2.81 (*n* = 133)	3.61 ± 2.56 (*n* = 99)	76.96 ± 8.34 (*n* = 133)

*HC* = healthy controls; *OB* = obesity; OTHER = dermatology + nephrology/BD; *RD* = respiratory diseases; *CKRL* = cumulative dysfunction score from the Computer Kinesiology system. Some n values differ between variables due to missing measurements (e.g., incomplete cortisol sampling or unavailable CKRL follow-up)

### Anthropometric and functional parameters

In the spa treatment group, the average BMI decreased by 0.7 points, with the most notable reductions observed in children with obesity. The Six-Minute Walk Test (6MWT) showed a 12% increase in the average walking distance, indicating an improvement in physical fitness. These changes were not statistically significant in the control group.

### Kinesiological and balance tests

Kinesiological assessment using the Computer Kinesiology system indicated improved functional status of the musculoskeletal system in 68% of children in the spa group. The Four Square Step Test (FSST) demonstrated enhanced dynamic balance and agility in 54% of these children. No significant changes were observed in these tests within the control group. Sex-stratified exploratory pre–post changes in 6MWT distance, FSST time, and CKRL score are summarized in Table 3.

**Table 3:** Within-group pre–post changes in 6MWT distance, FSST time and CKRL score by diagnostic group and sex (exploratory analysis)

Group	Sex	Variable	*n*	Stage 1 mean	Stage 2 mean	Δ (Stage 2 − Stage 1)	*t*-statistic	*p*-value	Cohen’s *d*
OB	Girls	6MWT distance (m)	21	494.57	531.71	+ 37.14	3.00	0.007	0.66
OB	Boys	6MWT distance (m)	15	543.67	563.60	+ 19.93	0.82	0.427	0.21
RD	Girls	6MWT distance (m)	24	630.33	654.33	+ 24.00	1.89	0.072	0.38
RD	Boys	6MWT distance (m)	26	629.50	642.92	+ 13.42	1.12	0.276	0.23
OB	Girls	FSST time (s)	21	9.06	8.03	− 1.03	− 5.47	< 0.001	− 1.19
OB	Boys	FSST time (s)	15	8.88	6.75	− 2.12	− 4.17	< 0.001	− 1.08
RD	Girls	FSST time (s)	24	9.75	7.65	− 2.09	− 2.66	0.014	− 0.54
RD	Boys	FSST time (s)	26	8.89	8.32	− 0.57	− 0.58	0.565	− 0.12
HC	Girls	FSST time (s)	20	8.24	7.75	− 0.49	− 1.40	0.178	− 0.30
HC	Boys	FSST time (s)	18	9.23	8.58	− 0.65	− 1.65	0.117	− 0.39
OB	Girls	CKRL score	21	89.38	73.43	− 15.95	− 6.60	< 0.001	− 1.44
OB	Boys	CKRL score	15	92.80	76.60	− 16.20	− 9.36	< 0.001	− 2.42
RD	Girls	CKRL score	24	80.42	75.79	− 4.63	− 1.96	0.063	− 0.40
RD	Boys	CKRL score	26	84.38	79.88	− 4.50	− 1.26	0.221	− 0.26
HC	Girls	CKRL score	20	81.86	80.95	− 0.90	− 0.55	0.589	− 0.12
HC	Boys	CKRL score	18	79.06	75.83	− 3.22	− 1.80	0.089	− 0.42

Δ = post–pre; values are means. *p*-values from paired *t*-tests; Cohen’s *d* = effect size for within-subject change. Data are exploratory and not adjusted for multiple comparisons; the study was not powered for sex-specific effects

### Correlations between parameters

Simple descriptive associations were examined to explore potential relationships between changes in salivary cortisol and key somatic or functional measures. In children with obesity, a decrease in morning cortisol tended to coincide with improvements in physical fitness (as reflected by the 6MWT) and with modest reductions in BMI. These parallel trends suggest that metabolic and functional adaptation may occur concurrently in this subgroup, although no causal interpretation can be drawn from these data.

Across the remaining diagnostic groups, changes in cortisol did not show a consistent association with BMI or functional performance. This variability indicates that cortisol changes may reflect diagnosis-specific physiological responses rather than a uniform pattern across the cohort. Figure 2 illustrates the distribution of BMI across diagnostic subgroups, including annotations of statistically significant within-group changes.

### Changes in salivary cortisol levels

In children with obesity, morning salivary cortisol decreased from 3.32 ± 2.05 to 2.36 ± 1.58 nmol/L (*n* = 34), with a paired mean change of − 0.96 nmol/L (95% CI − 1.54 to − 0.38; *p* = 0.004; Cohen’s *d* =  − 0.54). In children with chronic respiratory diseases without corticosteroid therapy, cortisol increased from 2.68 ± 0.97 to 3.48 ± 1.92 nmol/L (*n* = 18), corresponding to a mean change of + 0.80 nmol/L (95% CI − 0.02 to 1.62; *p* = 0.057; *d* = 0.47). Healthy controls showed an increase from 3.52 ± 2.70 to 4.02 ± 2.50 nmol/L (*n* = 36), with a mean change of + 0.50 nmol/L (95% CI − 0.08 to 1.09; *p* = 0.089; *d* = 0.25).

**Fig. 1:**
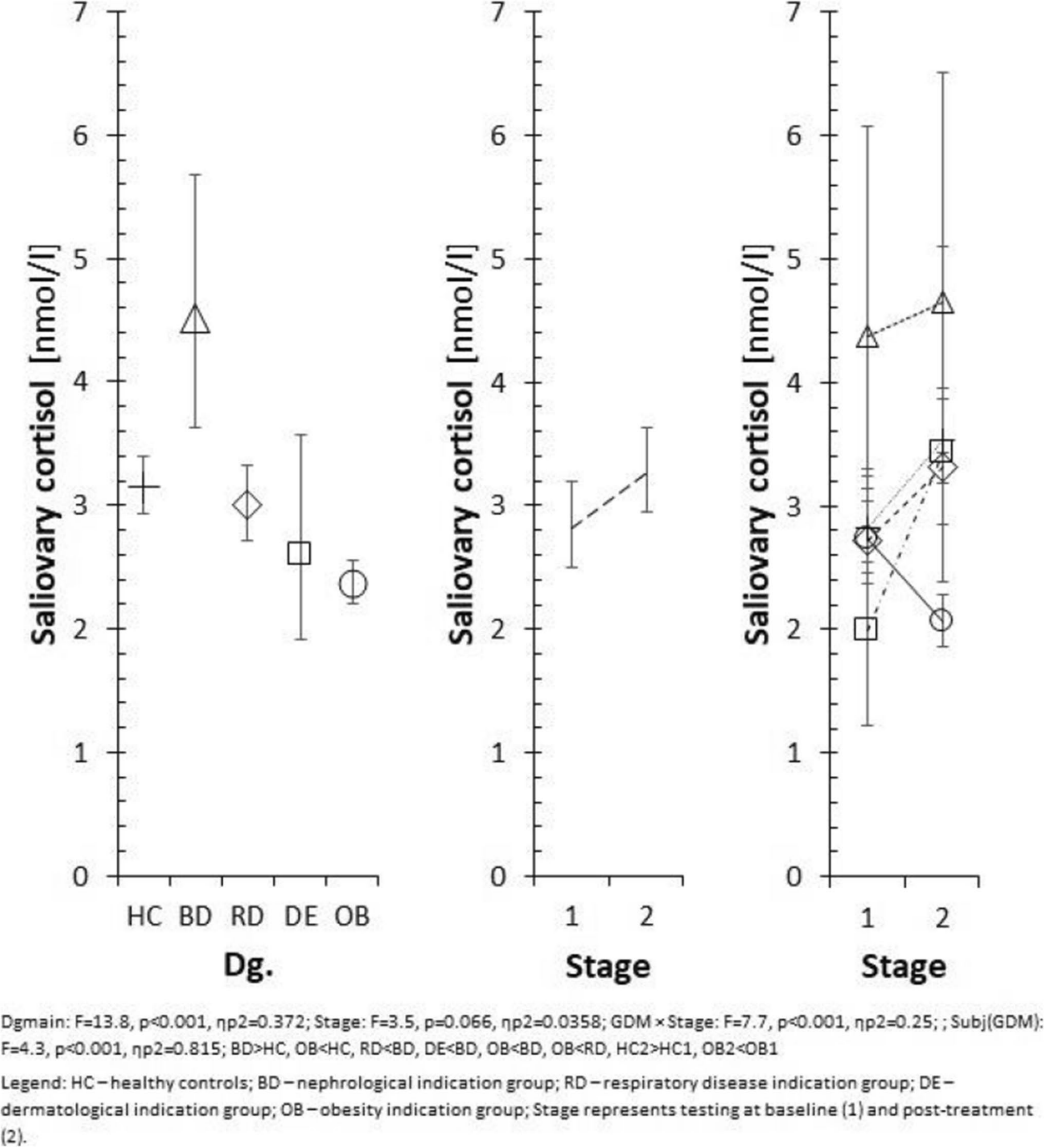
Salivary cortisol levels by diagnostic group. Dgmain: F = 13.8, *p* < 0.001, ηp^2^ = 0.372; Stage: F = 3.5, *p* = 0.066, ηp^2^ = 0.0358; GDM × Stage: F = 7.7, *p* < 0.001, ηp^2^ = 0.25; Subj(GDM): F = 4.3, *p* < 0.001, ηp.^2^ = 0.815. Pairwise contrasts: BD > HC, OB < HC, RD < BD, DE < BD, OB < BD, OB < RD, HC2 > HC1, OB2 < OB1. Legend: HC – healthy controls; BD – nephrological indication group; RD – respiratory disease indication group; DE – dermatological indication group; OB – obesity indication group. Stage represents testing at baseline (1) and post-treatment (2)

**Fig. 2:**
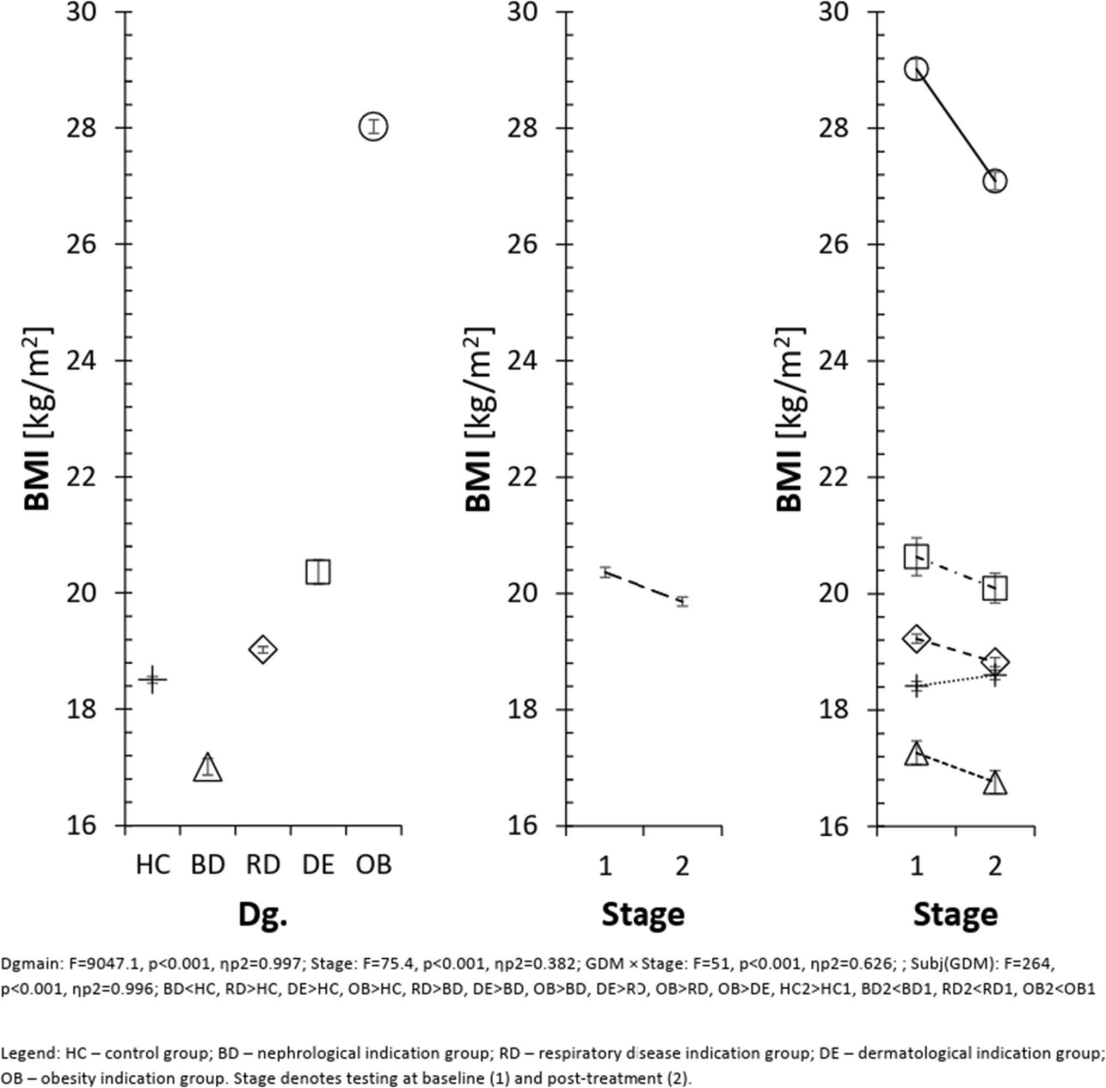
Body mass index (BMI) of individual participant groups according to primary diagnosis. Dgmain: F = 9047.1, *p* < 0.001, ηp^2^ = 0.997; Stage: F = 75.4, *p* < 0.001, ηp^2^ = 0.382; GDM × Stage: F = 51, *p* < 0.001, ηp^2^ = 0.626; Subj(GDM): F = 264, *p* < 0.001, ηp.^2^ = 0.996. Pairwise contrasts: BD < HC, RD > HC, DE > HC, OB > HC, RD > BD, DE > BD, OB > BD, DE > RD, OB > RD, OB > DE, HC2 > HC1, BD2 < BD1, RD2 < RD1, OB2 < OB1. Legend: HC – healthy controls; BD – nephrological indication group; RD – respiratory disease indication group; DE – dermatological indication group; OB – obesity indication group. Stage represents testing at baseline (1) and post-treatment (2)

Exploratory subgroup analyses did not reveal systematic sex-related differences in baseline cortisol or treatment-related changes, but the study was not powered to detect sex-specific effects. Figures 1 and 2 displays salivary cortisol levels across diagnostic groups.

A specific subgroup of children with chronic respiratory diseases (primarily bronchial asthma) was identified, in whom neither obesity nor corticosteroid therapy was present. This resulted in a comparably homogeneous subgroup without major confounding factors. Exploratory within-group pre–post changes in BMI and salivary cortisol stratified by sex are presented in Table 4.

**Table 4:** Within-group pre–post changes in BMI and salivary cortisol by diagnostic group and sex (exploratory analysis)

Group	Sex	Variable	*n*	Stage 1 mean	Stage 2 mean	Δ (Stage 2 − Stage 1)	*t*-statistic	*p*-value	Cohen’s *d*
OB	Girls	Salivary cortisol (nmol/L)	21	3.73	2.69	− 1.05	− 2.85	0.010	− 0.62
OB	Boys	Salivary cortisol (nmol/L)	15	2.66	1.86	− 0.81	− 2.64	0.021	− 0.70
RD	Girls	Salivary cortisol (nmol/L)	11	2.85	4.14	+ 1.29	2.09	0.063	0.63
RD	Boys	Salivary cortisol (nmol/L)	9	2.96	3.26	+ 0.30	0.58	0.579	0.19
Other	Girls	Salivary cortisol (nmol/L)	6	3.99	6.98	+ 2.99	1.83	0.127	0.75
Other	Boys	Salivary cortisol (nmol/L)	1	6.43	3.12	− 3.31	—	—	—
HC	Girls	Salivary cortisol (nmol/L)	20	3.58	4.09	+ 0.51	0.79	0.437	0.18
HC	Boys	Salivary cortisol (nmol/L)	18	3.58	4.21	+ 0.62	1.78	0.093	0.42
OB	Girls	BMI (kg/m^2^)	21	29.94	28.29	− 1.65	− 7.57	< 0.001	− 1.65
OB	Boys	BMI (kg/m^2^)	15	28.70	26.69	− 2.01	− 18.32	< 0.001	− 4.73
RD	Girls	BMI (kg/m^2^)	24	20.00	19.75	− 0.25	− 2.44	0.023	− 0.50
RD	Boys	BMI (kg/m^2^)	26	19.56	18.92	− 0.64	− 3.46	0.002	− 0.69
Other	Girls	BMI (kg/m^2^)	8	19.21	18.64	− 0.57	− 3.14	0.016	− 1.11
Other	Boys	BMI (kg/m^2^)	1	18.25	18.10	− 0.15	—	—	—
HC	Girls	BMI (kg/m^2^)	20	18.71	19.06	+ 0.34	2.92	0.008	0.64
HC	Boys	BMI (kg/m^2^)	18	19.11	19.23	+ 0.12	1.76	0.096	0.42

Δ = post–pre; *p*-values from paired *t*-tests; Cohen’s *d* = effect size for within-subject change. Data are exploratory and not adjusted for multiple comparisons; the study was not powered for sex-specific effects

## Discussion

In children with obesity, morning salivary cortisol showed a clear downward trend, consistent with studies reporting altered stress physiology associated with excess adiposity, low-grade inflammation, and irregular lifestyle patterns [14]. Because BMI in pediatric obesity is closely linked to metabolic and inflammatory load, even modest changes in weight may influence cortisol dynamics; overweight children often exhibit elevated or irregular cortisol rhythms that may normalize with improved physical activity and routine stabilization [4, 5]. Structured physical activity has also been associated with favorable cortisol and metabolic adaptation in overweight youth [15]. In our cohort, this pattern was accompanied by a statistically significant reduction in BMI and improvements in functional capacity, supporting the interpretation that morning cortisol in obese children may act as a sensitive marker of multifactorial behavioral and metabolic adaptation rather than an isolated endocrine signal.

In contrast, the mild increase observed in the respiratory subgroup aligns with evidence of atypical or paradoxical cortisol responses to physical or environmental stressors in pediatric asthma populations [6, 16]. These divergent trajectories are therefore not contradictory but rather suggest diagnosis-specific adaptive strategies under the same structured 28-day spa regimen. Given the pilot nature of this study, these interpretations remain tentative and require confirmation in controlled studies incorporating broader endocrine profiling.

At first glance, the opposite directions of change in obesity and respiratory groups might appear to question the usefulness of cortisol as a specific marker. However, these divergent trajectories are consistent with the concept of diagnosis-dependent HPA-axis adaptation under the same environmental load. In obesity, a downward shift in morning cortisol is compatible with partial normalization of a previously dysregulated stress system in the context of reduced metabolic load and more regular routines. In respiratory disease, a modest increase may reflect activation of a previously blunted axis in response to structured physical training and climatic stimuli, particularly in children with long-standing symptoms or prior corticosteroid exposure. Rather than indicating inconsistency, these findings suggest that salivary cortisol primarily captures the direction and magnitude of adaptation within each diagnostic context and should be interpreted in conjunction with disease characteristics and functional outcomes. A qualitative overview of these diagnostic trajectories is provided in Table 5.

**Table 5:** Qualitative summary of favorable pre–post changes by diagnostic group

Group	BMI	Cortisol	6MWT	FSST	CKRL
OB	Strong ↑	Strong ↑	↑	Strong ↑	Strong ↑
RD	→	Adaptive ↑	↑	↑	↑
OTHER	Mild ↑	Mixed	↑	↑	↑
HC	→	Mild adaptive ↑	n/a	→	→

Strong ↑ indicates a statistically significant favorable change with at least a moderate effect size (typically *p* ≤ 0.01 and approximately |*d*|≥ 0.5), clearly exceeding the magnitude of fluctuations observed in the HC group. Mild ↑ denotes a significant or borderline change with a small effect size (*p* ≤ 0.10 and |*d*|< 0.5) that is usually consistent with a modest improvement. For the RD group, however, the small reduction in BMI was statistically significant but not clearly favorable from a clinical perspective, as these children were not obese at baseline; therefore, this change is coded as → rather than Mild ↑. Adaptive ↑ describes a change toward an expected physiological range (for example, a modest cortisol increase in RD from low baseline levels) rather than deterioration. Mild adaptive ↑ in HC reflects small, non-significant shifts compatible with normal background variability in non-treated children. Mixed refers to heterogeneous patterns in small subgroups, and → indicates no relevant or statistically or clinically meaningful change beyond the variability observed in HC

The healthy control group served as a methodological reference to estimate natural variability in morning cortisol under everyday conditions. Routine psychosocial demands and behavioral factors in school settings are known to elevate cortisol independently of disease status [17, 18], and additional variability arises from non-standardized morning routines, including hygiene, food intake, or emotional state [19]. The mild increase observed in this group likely reflects normal physiological fluctuation. This interpretation is consistent with published normative ranges for morning salivary cortisol in healthy children, such as the interval of 1.7–12.8 nmol/L reported by Safarzadeh et al. [20]. Taken together, these data support the view that the changes observed in spa-treated children exceed the magnitude of ordinary day-to-day variability. Although BMI was not a primary explanatory variable in our models, the parallel decrease in BMI and cortisol in obese children, contrasted with stable BMI and rising cortisol in controls, argues against BMI acting as a simple confounder of cortisol dynamics in this setting.

Morning salivary cortisol appears particularly informative in children with obesity, where published evidence describes increased metabolic load, altered circadian regulation and higher variability of stress-related responses. In such children, cortisol may sensitively reflect multidimensional metabolic–behavioral adaptation to a structured environment, stable regimen, and supervised physical activity. In contrast, in respiratory conditions, interpretation of cortisol dynamics must consider respiratory effort, functional load, and environmental stimuli. Salivary cortisol should therefore not be regarded as a universal biomarker applicable uniformly across different pediatric diagnoses.

From a clinical perspective, salivary cortisol offers a practical, non-invasive means of assessing physiological reactivity to complex rehabilitative interventions. Its usefulness lies less in absolute values than in relative within-individual changes and diagnosis-specific trajectories. When interpreted alongside functional and somatic parameters, cortisol adds an important dimension to understanding neuroendocrine adaptation during spa treatment. Future studies should validate its role as part of an integrated biomarker panel, establish diagnosis-specific reference ranges, and examine its association with additional immune or autonomic markers.

This study intentionally focused on prepubertal children (9–12 years), a developmental period during which sex-related differences in morning cortisol are generally small. Exploratory comparisons within our cohort did not show consistent differences between boys and girls in baseline values or treatment-related changes. However, the sample size was insufficient to detect sex-specific effects with confidence, and larger cohorts will be required to explore potential sex-specific patterns further. Exploratory sex-stratified analyses (Table 4) did not reveal a consistent pattern of sex-specific effects, and these results should therefore be interpreted with caution.

Although the school-based comparison group is referred to as “healthy controls,” this designation does not imply medical verification. Rather, it reflects a convenience sample of school-aged children without acute illness or corticosteroid therapy. This population-based design provides a realistic reference frame for understanding natural cortisol dynamics in everyday life and supports the interpretation of treatment-related changes observed in the spa cohort.

## Limitations

This study has several limitations that should be considered when interpreting the findings. As a pilot prospective project, some diagnostic subgroups—particularly children with dermatological or nephrourological conditions—were small, and their values should be regarded as descriptive only and were not included in statistical comparisons.

The study intentionally focused on a prepubertal age range in which sex-related differences in morning cortisol are expected to be small. Although an exploratory comparison between boys and girls did not reveal systematic differences, the sample size was insufficient to draw firm conclusions about potential sex-specific patterns.

Another limitation relates to the different measurement environments. While procedures in the spa setting were largely standardized, the school-based control group was assessed under natural, everyday conditions, which inherently involve variation in morning routines, psychosocial factors and situational stress. This contextual difference introduces some variability, but it also provides a useful reference frame by illustrating the expected range of natural cortisol variability in routine daily life.

The analyses were not designed to evaluate individual components of the spa program separately, and it is therefore not possible to determine which aspects of the intervention contributed most to the observed changes. Similarly, the observed parallel changes between cortisol and functional outcomes should be interpreted as descriptive; the data do not permit causal inference.

Altogether, these limitations underscore the pilot nature of the study and the need for future research with larger cohorts, controlled designs, and additional biomarkers to further clarify the physiological mechanisms underlying treatment-related adaptation in children.

## Conclusion

This pilot study provides a real-world insight into diagnosis-specific changes in morning salivary cortisol in children undergoing a 28-day spa treatment program. A decrease in cortisol was observed in children with obesity, whereas a mild increase was seen in those with chronic respiratory conditions. These patterns suggest distinct physiological adaptations to the same structured therapeutic environment.

Salivary cortisol proved to be a practical, non-invasive indicator of physiological reactivity and complemented functional and somatic assessments, particularly in children with obesity where the observed decrease may reflect a favorable behavioral–metabolic adjustment to the spa regimen. In respiratory conditions, interpretation of cortisol dynamics requires careful consideration of functional load and environmental influences.

As an exploratory field-based study, these findings offer an initial framework for understanding hormonal adaptation during multidisciplinary spa treatment in children. They highlight the potential for differential treatment-related effects across diagnostic groups and may inform the focus and design of future research for investigators interested in this area.

## Data Availability

No datasets were generated or analysed during the current study.

## Abbreviations

BMI: Body mass index
DE: Dermatological Indication Group
FSST: Four Square Step Test
HPA axis: Hypothalamic–pituitary–adrenal axis
OB: Obesity Indication Group
RD: Respiratory Disease Indication Group
6MWT: Six-Minute Walk Test
HC: Healthy controls
